# Regulatory and memory T lymphocytes infiltrating prostate tumors predict long term clinical outcomes

**DOI:** 10.3389/fimmu.2024.1372837

**Published:** 2024-06-03

**Authors:** Oscar Eduardo Molina, Hélène LaRue, David Simonyan, Hélène Hovington, Benjamin Vittrant, Bernard Têtu, Vincent Fradet, Louis Lacombe, Alain Bergeron, Yves Fradet

**Affiliations:** ^1^ Axe oncologie, Centre de recherche du CHU de Québec-Université Laval, Québec, QC, Canada; ^2^ Centre de recherche sur le cancer de l’Université Laval, Québec, QC, Canada; ^3^ Plateforme de recherche clinique et évaluative, Centre de recherche du CHU de Québec-Université Laval, Québec, QC, Canada; ^4^ Département de pathologie, CHU de Québec-Université Laval, Québec, QC, Canada; ^5^ Département de chirurgie, Université Laval, Québec, QC, Canada

**Keywords:** prostate cancer, clinical outcomes, prognosis biomarkers, immunohistochemistry, tumor immune cell infiltration, lymphocytes, regulatory T cells, memory T cells

## Abstract

**Introduction:**

The localization, density but mostly the phenotype of tumor infiltrating lymphocytes (TIL) provide important information on the initial interaction between the host immune system and the tumor. Our objective was to assess the prognostic significance of T (CD3^+^), T regulatory (T_reg_) (FoxP3^+^) and T memory (T_mem_) (CD45RO^+^) infiltrating lymphocytes and of genes associated with TIL in prostate cancer (PCa).

**Methods:**

Immunohistochemistry (IHC) was used to assess the infiltration of CD3^+^, FoxP3^+^ and CD45RO^+^ cells in the tumor area, tumor margin and adjacent normal-like epithelium of a series of 98 PCa samples with long clinical follow-up. Expression of a panel of 31 TIL-associated genes was analyzed by Taqman Low-Density Array (TLDA) technology in another series of 50 tumors with long clinical follow-up. Kaplan-Meier and Cox proportional hazards regression analyses were performed to determine association of these markers with biochemical recurrence (BCR), need for definitive androgen deprivation therapy (ADT) or lethal PCa.

**Results:**

TIL subtypes were present at different densities in the tumor, tumor margin and adjacent normal-like epithelium, but their density and phenotype in the tumor area were the most predictive of clinical outcomes. In multivariate analyses, a high density of T_reg_ (high FoxP3^+^/CD3^+^ cell ratio) predicted a higher risk for need of definitive ADT (HR=7.69, p=0.001) and lethal PCa (HR=4.37, p=0.04). Conversely, a high density of T_mem_ (high CD45RO^+^/CD3^+^ cell ratio) predicted a reduced risk of lethal PCa (HR=0.06, p=0.04). TLDA analyses showed that a high expression of FoxP3 was associated with a higher risk of lethal PCa (HR=5.26, p=0.02). Expression of CTLA-4, PD-1, TIM-3 and LAG-3 were correlated with that of FoxP3. Amongst these, only a high expression of TIM-3 was associated with a significant higher risk for definitive ADT in univariate Cox regression analysis (HR=3.11, p=0.01).

**Conclusion:**

These results show that the proportion of T_reg_ and T_mem_ found within the tumor area is a strong and independent predictor of late systemic progression of PCa. Our results also suggest that inhibition of TIM-3 might be a potential approach to counter the immunosuppressive functions of T_reg_ in order to improve the anti-tumor immune response against PCa.

## 1 Introduction

Prostate cancer (PCa) remains the second most commonly diagnosed cancer and the fifth cause of cancer death among men worldwide despite PCa screening and early effective treatments (1, 2). This may be due in part to the late systemic recurrences occurring after years of apparent PCa control in aging men who live longer, thanks to reduced competing cause of mortality. The management of PCa is challenging because this cancer is a highly heterogeneous disease (3, 4). Therapeutic options will vary considerably for patients with low-risk indolent PCa and those with high-risk life-threatening PCa. One major challenge lies in the adequate risk stratification to help select the most appropriate therapeutic strategy and avoid under or overtreatment (5). So far, the stratification of risk is based on clinico-pathological factors such as tumor grade (Gleason score) and stage (TNM), PSA level at diagnosis and presence of adverse pathological features at prostatectomy (6, 7). However, the risk of recurrence and progression and the response to treatments vary significantly between patients with otherwise similar clinico-pathological characteristics. Therefore, new biomarkers are needed to refine the prognostication and improve the management of PCa (8). Moreover, the most common endpoint of biomarker studies in PCa has been biochemical recurrence (BCR), but few studies have been able to relate biomarkers with long-term outcomes such as metastasis and mortality by PCa. It is even more challenging to identify biomarkers in the primary PCa that could lead to potential early interventions to reduce late PCa mortality.

The influence of the tumor microenvironment (TME) on the development and progression of cancer has gained greater interest during the last decade (9). The TME influences cancer evolution through diverse mechanisms including tumor differentiation, stimulation of angiogenesis and promotion of immune evasion (10–12) Tumor-infiltrating lymphocytes (TIL) participate in the host defense against tumor cells but their antitumor activity is greatly hampered by the immunosuppressive factors found within the TME which favors immune evasion (13). Several studies have shown that the density, phenotype and localization of TIL are associated with clinical outcomes in various types of solid cancers and that their detailed analysis can provide important prognostic information (14, 15). The prognostic potential of TIL has been notably well demonstrated in colorectal cancers (CRC). Studies by Galon et al. have shown that TIL could predict better than TNM disease-free survival and overall survival of CRC patients (16, 17). These studies led to the development of the “Immunoscore” to complement the current TNM in order to better predict the risk of recurrence and even response to therapy of CRC patients (18–20).

While the prognostic potential of TIL has been well established in CRC and some other solid cancers, the study of the relationship between TIL and the clinical outcomes of PCa has provided inconsistent results (21–33). These inconsistencies may be due in part to differences in study designs, to various subtypes of TIL studied and their localization within the specimen and to the different methodologies used to measure them. The types of patients’ cohorts and the length of their follow-up are also very important parameters to assess the clinical relevance of the findings. The most recent immunohistochemical (IHC) studies have focused on regulatory T (T_reg_) cells using antibodies against the transcription factor FoxP3, the most specific biomarker of the T_reg_ cells. These studies showed an association between FoxP3^+^ TIL and higher risk of BCR or death from PCa (25, 31, 34). However, very few studies have characterized the infiltration of prostate tumors by CD45RO^+^ cells. CD45RO is a marker of central and effector memory T (T_mem_) cells and is therefore associated with an effective immune response. In CRC, the combined analysis of CD45RO^+^ T_mem_ cells and CD8^+^ T cells in specific tumor areas helped predict cancer recurrence and patients’ survival (17).

To further characterize the intra-tumoral immune response against PCa and assess the potential prognostic value of TIL, we analyzed the infiltration of CD3^+^, FoxP3^+^ and CD45RO^+^ cells in the tumor core, tumor margin and the adjacent normal-like epithelium area in radical prostatectomy specimens from 98 PCa patients at increased risk of recurrence and with very mature long-term clinical follow-up. We also analyzed the expression of 31 genes associated with TIL by qRT-PCR using TaqMan^®^ Low Density Array (TLDA) technology in another set of 50 similar patients with fresh frozen radical prostatectomy. The combined results show that the balance between T_mem_ and T_reg_ infiltrating cells within the cancer is a strong independent predictor of systemic cancer progression and lethality by PCa. They also identify the immune checkpoint TIM-3 as a potential target to reverse T_reg_ dominant immunosuppression in PCa.

## 2 Results

### 2.1 Immunohistochemistry analyses

#### 2.1.1 Cohort description

**Figure 1:**
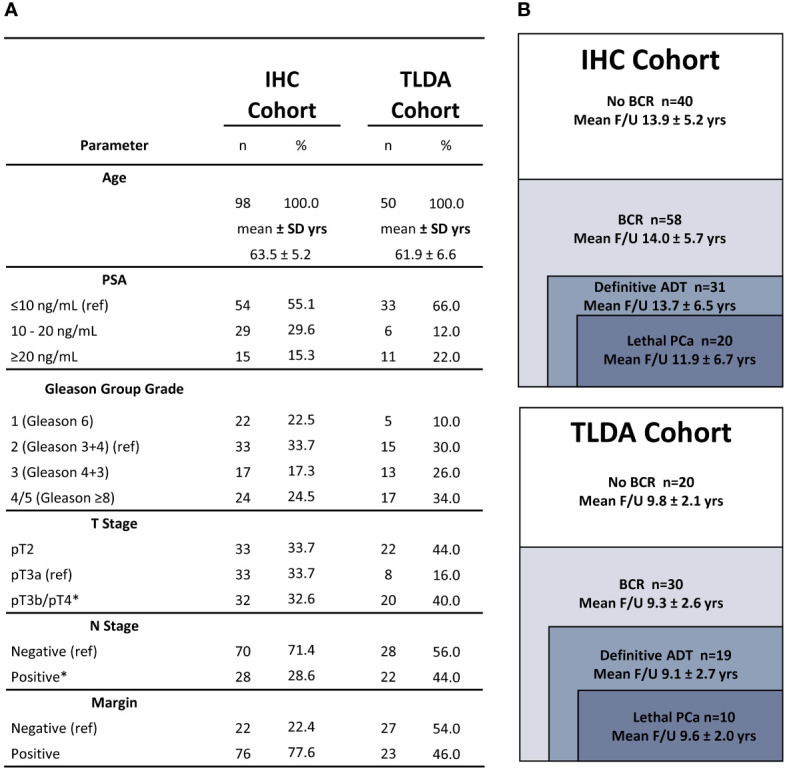
Cohort description. **(A)** Baseline characteristics of the cohorts used for IHC or TLDA analyses. The number of patients in each category is provided. The mean age is indicated in years (yrs). **(B)** Mean follow-up of the cohorts used for IHC or TLDA analyses according to each clinical outcome. Asterisks indicate clinicopathologic factors significantly associated with definitive ADT and lethal PCa in multivariate Cox regression analyses. Variables of reference are indicated by: (ref).

The infiltration by CD3^+^, CD45RO^+^ and FoxP3^+^ lymphocytes was analyzed in a series of 98 prostatectomy specimen of PCa patients at increased risk of recurrence and progression and with a very long clinical follow-up (median of 15.5 years; mean of 14.0 years). **Figure 1** shows the baseline characteristics of this cohort (IHC cohort). The long clinical follow-up of these tumors allows for a more accurate association of the markers with late clinical events. These events were defined as occurrence of a BCR, the need for continuous androgen-deprivation therapy (ADT) and lethal cancer defined as having a metastatic disease and/or castration-resistant PCa (CRPC) and/or death from PCa. Multivariate Cox regression analyses were performed to assess the association between the age, PSA levels, Gleason Group Grade categories, stage categories and presence or not of positive surgical margins with each of the clinical endpoints. **Supplementary Table S1** shows that pT3b/pT4 and lymph node invasion were independent predictors of definitive ADT and lethal PCa. Moreover, in this cohort, from the 98 patients, 58 experienced BCR at a median time of 6 years: 31 progressed to continuous ADT and 20 to lethal PCa. Of the other 27 BCR patients who did not progress to ADT or lethal PCa, 21 responded to salvage radiotherapy and 6 received short-term intermittent ADT.

#### 2.1.2 Immunohistochemistry scoring

**Figure 2:**
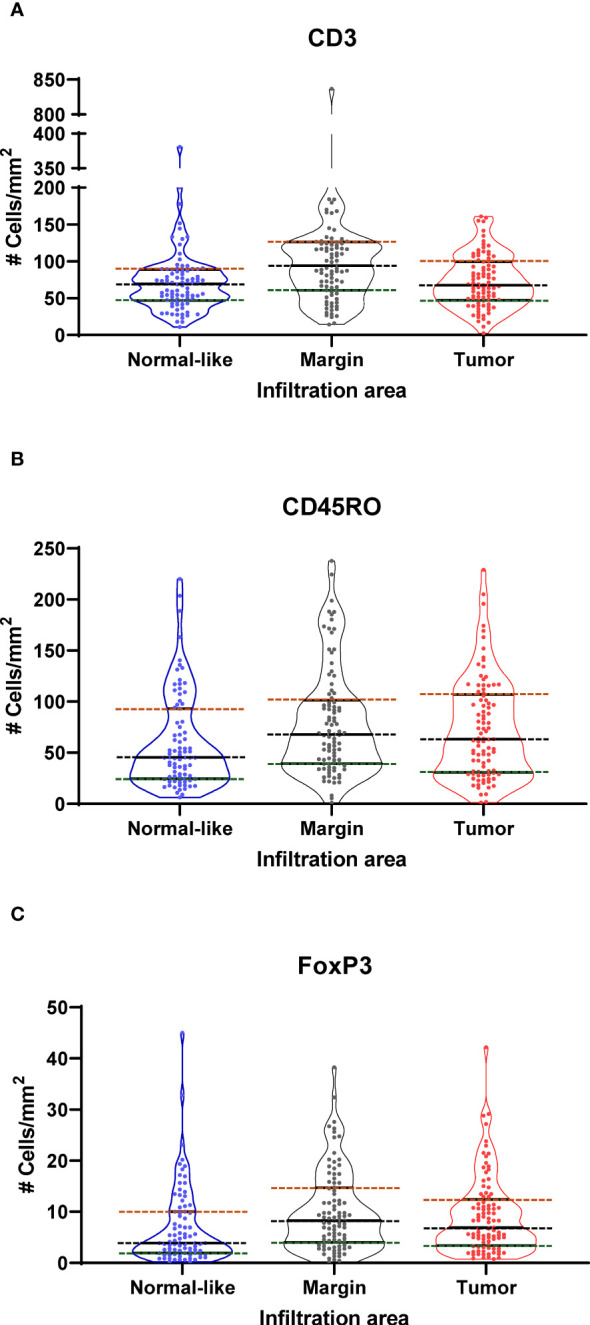
Violin plots showing the distribution of the density (cells/mm^2^) of **(A)** CD3^+^ **(B)** CD45RO^+^ and **(C)** FoxP3^+^ cells in normal-like adjacent epithelium, tumor margin and tumor areas as determined by immunohistochemistry analysis of 98 prostate cancer specimens. Dotted lines show boundaries of Q1 (green), Q2 or median (black) and Q3 (red) quartiles.

The number of positive cells per mm^2^ was determined in the tumor area, at the tumor margin and in the adjacent normal-like epithelium (**Supplementary Figure S1**). Positive cells in tertiary lymphoid aggregates were not considered in the analysis. The evaluation of the infiltration in the tumor and the margin areas showed that the highest density of these positive cells was found at the tumor margin. A qualitative description of the infiltration for each type of positive cells is provided in **Supplementary Data**. The distribution of cell densities for each TIL subtype, in each compartment and for each patient is represented in **Figure 2** with bars separating the data into quartiles. Examples of the staining obtained with the antibodies against CD3, CD45RO and FoxP3 are shown in **Supplementary Figure S2**.

#### 2.1.3 Association with clinical outcomes

Kaplan-Meier analyses were performed to determine the association between the levels of infiltration by each type of immune cells in each of the three compartments and the clinical outcomes. Kaplan-Meier analyses were performed with data categorized into quartiles and dichotomized as the lowest quartile *vs* the three other quartiles (Q1, *i.e.* low infiltration *vs* Q2-Q4) or as the highest quartile *vs* the three other quartiles (Q4, *i.e.* high infiltration *vs* Q1-Q3). Ratios between markers were determined for each compartment. The ratios were divided into quartiles to be analyzed by Kaplan-Meier curves and in multivariate proportional hazards Cox regression analyses. The ratios of FoxP3^+^ and CD45RO^+^ cells over the CD3^+^ cells in the tumor area as well as the ratios of FoxP3^+^ over the CD45RO^+^ cells in the tumor area were significantly associated with clinical outcomes.

**Figure 3:**
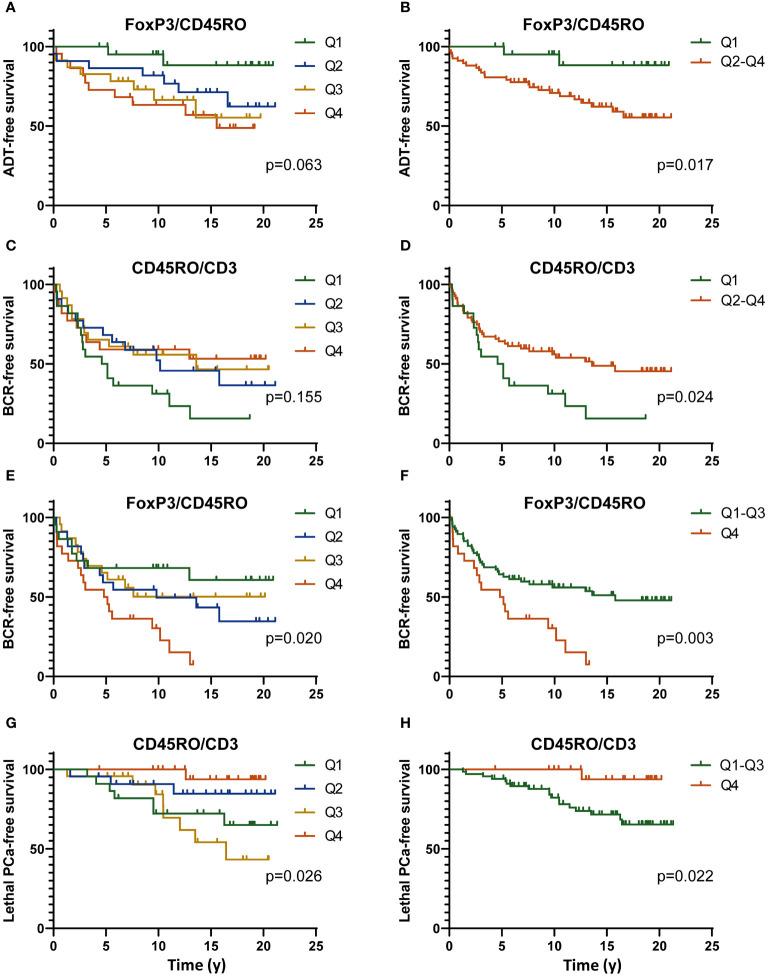
Kaplan-Meier curves showing the definitive ADT-free **(A, B)**, BCR-free **(C–F)** or lethal PCa-free survival according to the level of the ratio of the number of FoxP3^+^/CD45RO^+^ cells **(C–F)** or CD45RO^+^/CD3^+^ cells **(C, D, G, H)** categorized as quartiles (Q1 to Q4) **(A, C, E, G)** or dichotomized as to low value vs high (Q1 vs Q2-Q4) **(A–D)** or high value vs low (Q4 vs Q1-Q3) **(E–H)** of the ratios. All data are from the analysis of the infiltration of these immune cells in the tumor area only. The *p* value were estimated by the log-rank test.

Some examples of the Kaplan-Meier curves comparing the levels of the ratio of FoxP3^+^/CD45RO^+^ cells categorized as quartiles or dichotomized as Q1 *vs* Q2-Q4 or Q1-Q3 *vs* Q4 and of the ratio of CD45RO^+^/CD3^+^ cells categorized as quartiles or dichotomized as Q4 *vs* Q1-Q3 or Q1-Q3 *vs* Q4 for their association with outcomes are presented in **Figures 3A–H**. **Figure 3B** shows that a low ratio (Q1) of FoxP3^+^/CD45RO^+^ cells was associated with a longer survival without the need for definitive ADT (log-rank p=0.017) while **Figure 3F** shows that a high ratio (Q4) of FoxP3^+^/CD45RO^+^ cells predicts a shorter time to BCR (log-rank=0.003). At the opposite a low ratio (Q1) of CD45RO^+^/CD3^+^ is associated with a shorter BCR-free survival (**Figure 3D**, log-rank=0.024) while a high ratio (Q4) of CD45RO^+^/CD3^+^ cells was associated with a longer lethal PCa-free survival (**Figure 3H**, log-rank=0.022).

The association between the levels of these cell densities or the ratios of the cell density and the clinical outcomes was further studied using univariate and multivariate Cox regression analyses. Univariate Cox models revealed several associations with outcomes but since the objective was to assess the independent prognostic value of TIL, only results from multivariate analyses are presented here. While looking at the whole population of lymphocytes, we found that high density of CD3^+^ in the adjacent normal-like epithelium or at the margin was associated with a lower risk of BCR (HR=0.45, p=0.04 and HR=0.36, p=0.03, respectively). On the other hand, a low density of CD3^+^ cells in the tumor area was associated with a higher risk of lethal PCa (HR=3.77, p=0.04).

**Figure 4:**
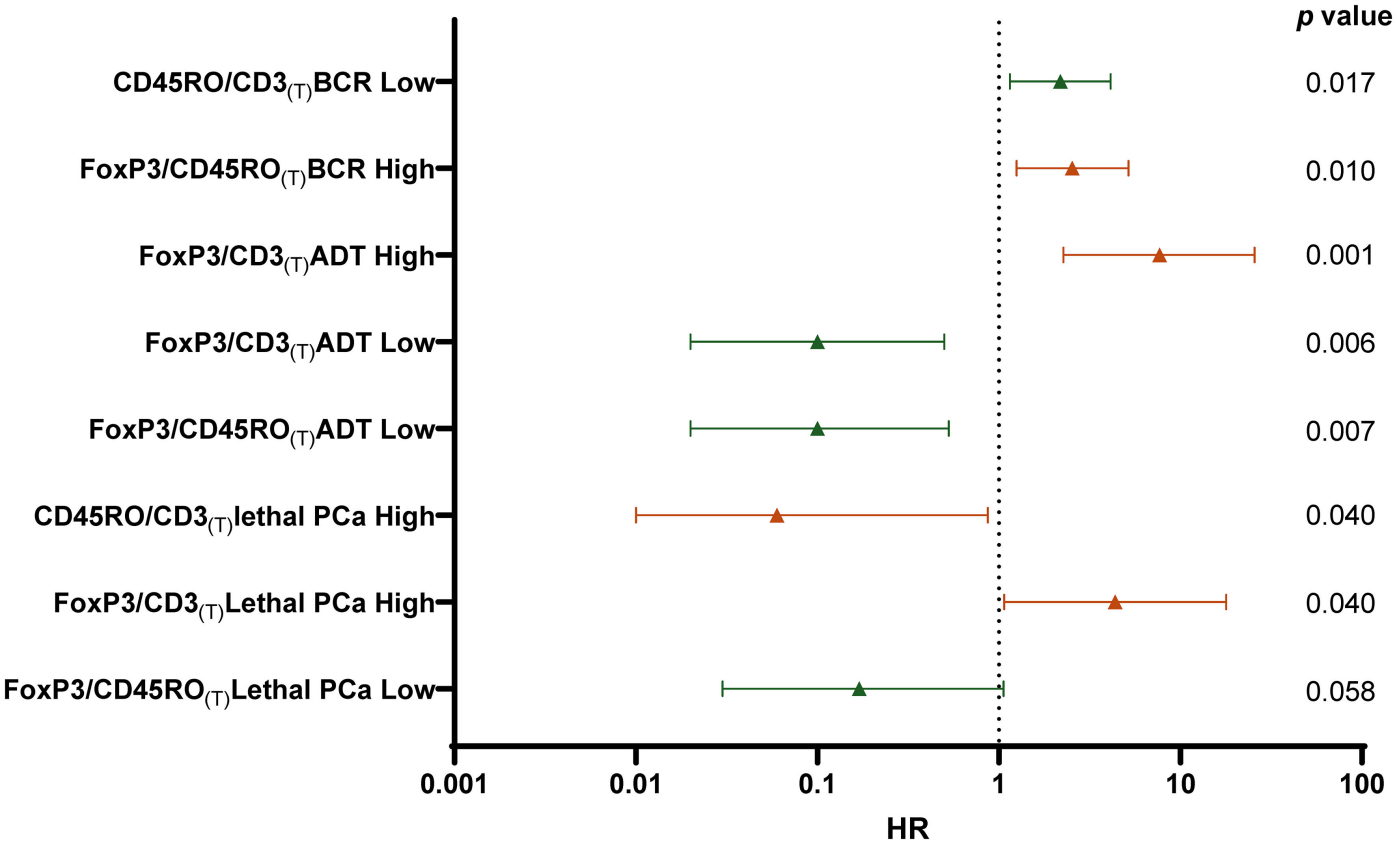
Forest plot of multivariate Cox regression analysis calculated HR to predict the risk for each clinical outcomes (BCR, ADT or Lethal PCa) according to low density (Low -green) (Q1 vs Q2-Q4) or high density (High- red) (Q4 vs Q1-Q3) ratio of the number of cells infiltrating the tumor area (T). HR was adjusted for age, PSA, Gleason grade, T stage, N stage, and margin status. The p values are indicated at the right of the forest plot.

The ratios of FoxP3^+^/CD3^+^, CD45RO^+^/CD3^+^ and the FoxP3^+^/CD45RO^+^ provided several significant associations with the outcomes (**Supplementary Table S2**). Interestingly, it was mostly the ratio of the cell densities within the tumor area that were found to be significantly associated with the clinical events. **Figure 4** shows in a forest plot the various HR values and corresponding p values for the ratio of these cell populations that had the strongest association with the outcomes. As presented, a high ratio of FoxP3^+^/CD3^+^ cells was associated with higher risk (HR=7.69, p=0.001) whereas at the opposite a low ratio of FoxP3^+^/CD3^+^ cells was associated with a lower risk (HR=0.10, p=0.006) of the need for definitive ADT. Consistent with this result, a high ratio of FoxP3^+^/CD3^+^ cells was also associated with a higher risk of lethal PCa (HR=4.37, p=0.040). Also, a high ratio of FoxP3^+^/CD45RO^+^ cells was associated with a higher risk (HR=2.54, p=0.010) of BCR whereas a low ratio was associated with a lower risk of needing definitive ADT (HR=0.10, p=0.007) and also with a lower risk of lethal PCa (HR=0.17, p=0.058), although the statistical significance was not reached. Finally, a low ratio of CD45RO^+^/CD3^+^ cells was associated with a higher risk (HR=2.18, p=0.017) of BCR whereas a high ratio was associated with a lower risk (HR=0.06, p=0.040) of lethal PCa.

### 2.2 Gene expression analyses

#### 2.2.1 Cohort description

A total of 50 frozen radical prostatectomy specimens were available for the analysis of immune genes. These tumors were selected in order to have a representation of tumor and peri-tumor tissues including normal-like glands as we wanted to include in the analysis immune cells from the peri-tumor area since these might have important prognosis value. As for the IHC cohort, this cohort also contains a high proportion of tumors at higher risk of recurrence and progression and has a distribution of clinical outcomes that is similar to that of the IHC cohort (**Figure 1**). Despite a shorter < 10 years mean follow-up, a similar proportion *i.e*. 30 out of 50 patients experienced BCR and 19 progressed to ADT of which 10 progressed to lethal PCa. Similarly, for the 11 BCR patients who did not progress to ADT or lethal PCa, 9 responded to salvage radiotherapy and 2 received short-term ADT.

#### 2.2.2 FoxP3 is associated with lethal PCa

The RNA extracted from these tumors was tested using TLDA for the expression of 31 genes associated with T lymphocyte phenotypes and functions (**Supplementary Table S3**). The level of expression was normalized over the expression of two housekeeping genes, *i.e.* GUSB and PPIA.

**Figure 5:**
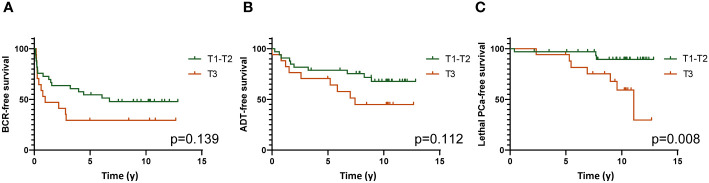
Kaplan-Meier survival analyses showing the association of high (Tertile 3) vs low (Tertiles 1 and 2) expression of FoxP3 gene with survival without BCR **(A)**, definitive ADT **(B)** and lethal PCa **(C)**. Log-rank test was used to determine the *p* values.

Relative quantification values for each gene were categorized as tertiles (T1 to T3). Data dichotomized as high (T3) *vs* low (T1-T2) expression were analyzed in function of the clinical outcomes using the Kaplan-Meier estimator. This analysis revealed that very few genes were associated with the outcomes. Among these was FoxP3. **Figure 5C** shows that a high expression (T3) of FoxP3 was associated with a shorter lethal PCa-free survival (log-rank p=0.008). High *vs* low expression of FoxP3 was however not able to significantly predict the survival without ADT nor BCR although a clear separation of the curves can be observed (**Figures 5A, B**).

The association of high expression of FoxP3 with a higher risk of lethal PCa was also observed in univariate Cox regression analysis (HR=5.26, 95%CI=1.35-20.41, p=0.017). The expression of this gene was however not predictive of the survival without ADT or BCR (**Supplementary Table S4**).

#### 2.2.3 Expression of FoxP3 gene correlates with that of CTLA-4

**Figure 6:**
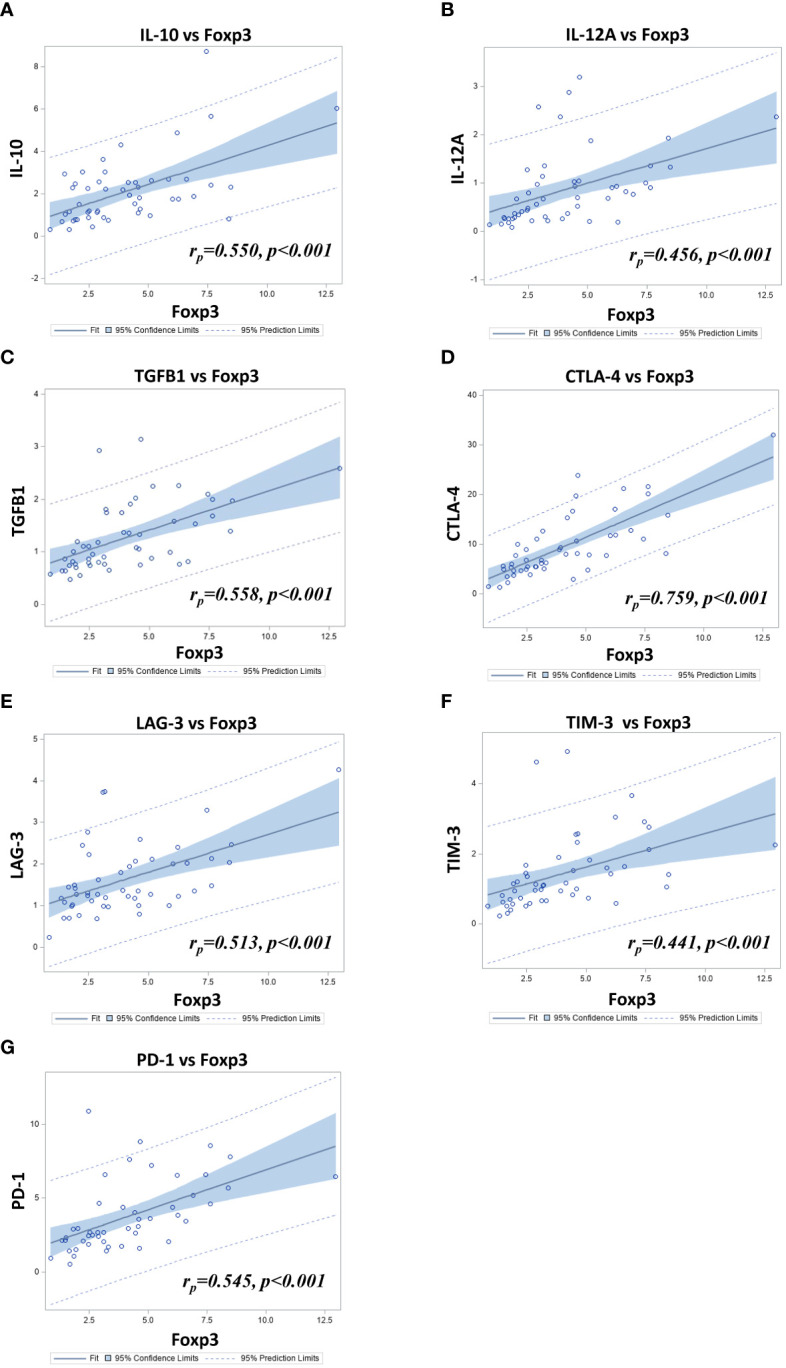
Scatter-plots combined with fit-plots for the Pearson correlation between FoxP3 and **(A)** IL-10 (IL10), **(B)** IL-12A (IL12A), **(C)** TGFβ1 (TGFB1) **(D)** CTLA-4 (CTLA4) **(E)** LAG-3 (LAG3) **(F)** TIM-3 (HAVCR2) and **(G)** PD-1 (PDCD1) relative gene expression in 50 PCa samples as determined by TLDA.

We performed a Pearson correlation with each gene of the T_reg_ and the immune checkpoint (ICP) pathway from our selected targets to determine which genes where the most strongly correlated with FoxP3. CTLA-4 was among the genes that were the most strongly correlated with FoxP3 *(r_p_=0.759, p<0.001)*. Since T_reg_ exert their immunosuppressive activity through secretions of cytokines such as TGFß1, IL-10 and IL-35, we also looked at the correlation of FoxP3 with the genes encoding these cytokines. As IL-35 is a cytokine that is part of the IL-12 cytokine family and is composed of two subunits, *i.e.* IL-12α and IL-27β chains, we only correlated FoxP3 with IL-12A as the gene encoding IL-27β was not in our panel. There was a significant correlation of FoxP3 expression with that of IL-10 *(r_p_=0.550, p <0.001)*, IL-12A *(r_p_= 0.456, p<0.001)* and TGFß1 *(r_p_=0.558, p<0.001)* (**Figure 6**).

**Figure 7:**
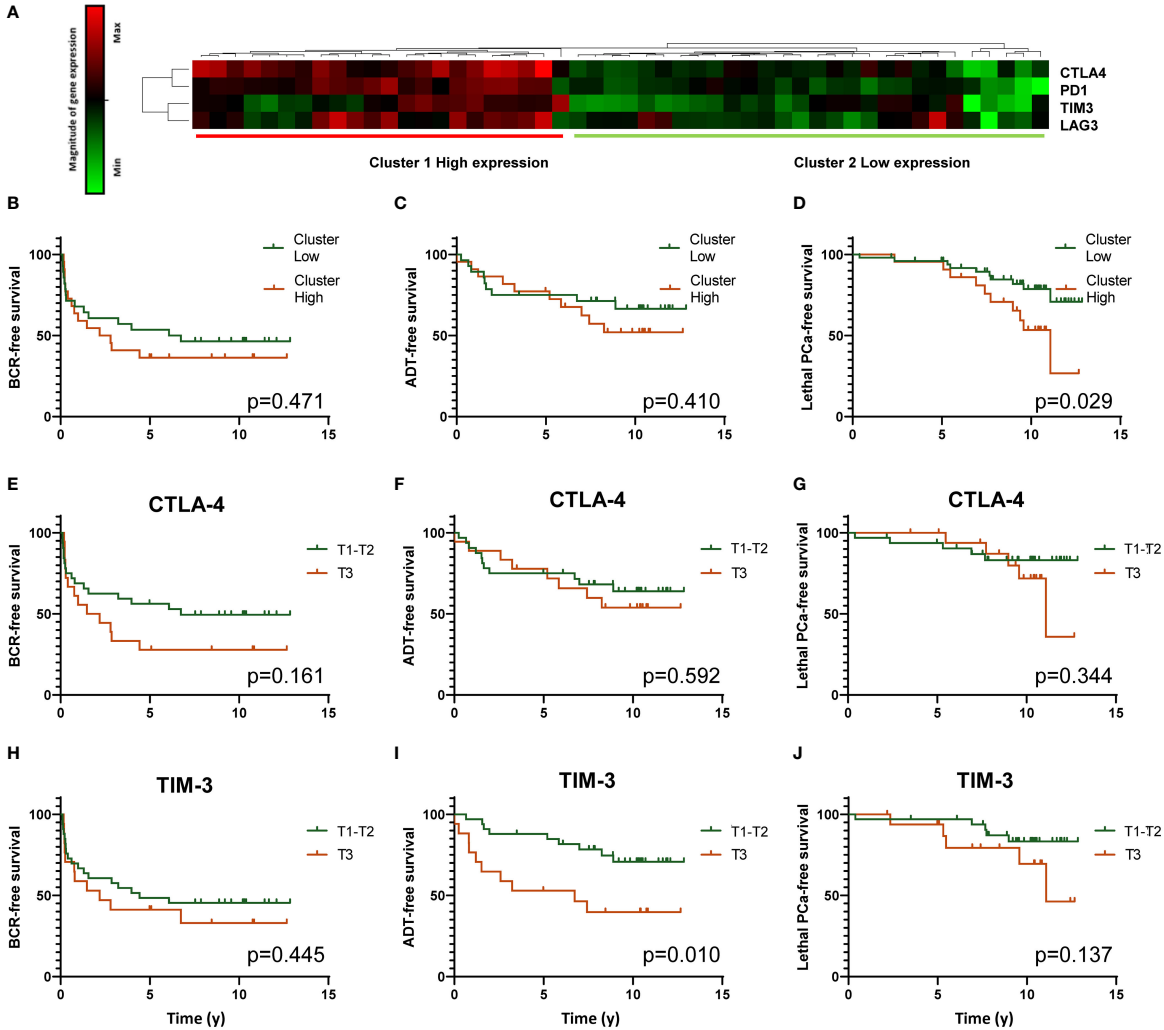
Analysis of CTLA-4 (CTLA4), PD-1 (PDCD1), TIM-3 (HAVCR2) and LAG-3 (LAG3) gene expression by TLDA. Heatmap resulting from the unsupervised hierarchical clustering of the expression of four ICP genes into two clusters corresponding to high and low expression of the genes is presented **(A)**. Gene expression determined by real time qPCR with Taqman probes is shown in the heatmap. Each horizontal row represents the same gene product and each vertical row, each patient. Magnitude of expression from high (red) to low (green) is indicated by the colored bar. Kaplan-Meier survival curves showing the association of high (red) and low (green) expression clusters with survival without BCR, definitive ADT and lethal PCa are presented in **(B–D)** panels, respectively. Other Kaplan-Meier analyses were performed to analyse the association between individual ICP gene expression and the three clinical outcomes. Panels **(E–G)**, **(H–J)** present these analyses for CTLA-4 and TIM-3, respectively. A high expression of TIM-3 is significantly associated with a shorter survival without definitive ADT. Log-rank test was used to determine the *p* value.

In view of the importance of ICP in the function of TIL, and since we found a significant correlation between the expression of FoxP3 and CTLA-4, we further looked at the correlation between the expression of FoxP3 with the expression of other important ICP. LAG-3*, (r_p_=0.513, p <0.001)*, TIM-3 *(r_p_= 0.441, p<0.001)* and PD-1 *(r_p_=0.545, p<0.001)* were all found to be correlated with FoxP3 (**Figure 6**). In order to assess the correlation between the expression of these ICP and the outcomes, a hierarchical clustering was performed to define two molecular subgroups based on the expression of the 4 genes (low *vs* high expression) (**Figure 7A**). Kaplan-Meier survival curves were used to determine the association of these two groups with clinical outcomes. Results showed that tumors showing a high expression of these genes were associated with a shorter lethal PCa-free survival (log-rank p=0.029)(**Figure 7D**), but they were not significantly associated with survival without BCR nor ADT (**Figures 7B, C**). Kaplan-Meier analyses were also conducted with data from these genes taken individually (**Figures 7E–J**). **Figure 7E** shows that a high expression of CTLA-4 (T3) tended to be associated with a shorter BCR-free survival but the difference between the curves were not statistically significant. However, a high expression of TIM-3 (T3) was predictive of a shorter ADT-free survival (log-rank p=0.010) (**Figure 7I**) but not of BCR and lethal PCa-free survivals (**Figures 7H, J**). This association of TIM-3 with late clinical outcomes was also observed in Cox regression analysis as a high expression of TIM-3 (T3) was associated with higher risk of definitive ADT (HR=3.11 (1.25-7.69), p=0.014; **Supplementary Table S4**). High expression of LAG3 or PD-1 was not significantly associated with any of the outcomes in these analyses.

## 3 Discussion

T (CD3^+^) and B (CD20^+^) lymphocytes infiltrate prostate cancers, but it was previously shown that B lymphocytes are not associated with clinical outcomes (24). We thus deliberately omitted the analysis of B lymphocytes in this study. CD3^+^ TIL are the effectors of the adaptative cellular immune response and therefore their number, phenotype and localization provide important information on the antitumor response specifically directed against tumor antigens and have been shown to predict clinical outcomes in many types of cancers (19, 35–37). Their presence reflects the immunogenicity of the tumor caused by the presence of either shared tumor antigens or mutated antigens. Although the mutational burden of most PCa is low compared to melanomas or lung cancers, some PCa show some high levels of TIL which might reflect some specific characteristics of these tumors (38, 39). It has been notably shown that tumor with deficient mismatch repair mechanisms are associated with a higher density of TIL due to the creation of neoepitopes resulting from the unrepaired DNA replication errors (40). Similarly, it was found that advanced PCa with inactivating CDK12 mutations, which affect the expression of genes involved in DNA damage response, had elevated neoantigen burden and were more infiltrated with T cells which even showed some clonal expansion (41, 42). Other characteristics were associated with a higher density of TIL in PCa. Vidotto et al. and Kaur et al. showed that tumors with loss of the PTEN tumor suppressor gene had higher density of FoxP3^+^ cells (33, 43). It was also found that higher TIL density was more frequent in ERG positive tumors (24, 33, 44). Hence, there is growing evidence of a relationship between genomic alterations and immune cell infiltration in PCa and thus the level of TIL subtypes may reflect distinct biological behaviors and prognoses.

In this study, we analyzed the infiltration of TIL by immunohistochemistry using CD3 as a marker to identify the entire T cell population, FoxP3 to identify the T_reg_ cells and CD45RO to identify T cells with a memory phenotype. We studied these T cell subtypes in three compartments of the tissue sections in order to assess whether the localization of these immune cells had a significant impact on the prognosis. Contrary to many other immunohistochemical studies that analyzed TIL using tissue microarrays (TMA) (22–25, 31, 33, 34) our study was performed on whole sections of FFPE tumors which allowed us to assess more accurately the number of TIL in the different tissue compartments. We showed that no matter the phenotype, the number of CD3^+^, FoxP3^+^ or CD45RO^+^ cells was highly variable from one tumor to the other and these cells were on average more frequent at the tumor margin, although the difference was not statistically significant (**Figure 2**). This is consistent with another study reported by Yuan et al. which found that the majority of immune cells were found in the normal-like or pre-invasive tissue areas rather than in the tumor core (45). The higher density of immune cells at the tumor margin may reflects the properties of the tumor microenvironment making access to the tumor core more difficult. Such accumulation of immune cells outside the tumor core might provide important prognostic information. However, we found that it was the density of the T cells within the tumor area and not in the peritumor area (margin and adjacent normal-like areas) that was the most informative on the late clinical outcomes suggesting that T cells within the tumor core have a more important impact on the tumor evolution (**Figure 4**, **Supplementary Table S2**).

Our results showed that a high density of CD3^+^ cells in the peritumor area was associated with a lower risk of BCR while a low density of these cells in the tumor area increased the risk of lethal PCa. This is in contrast with some studies that concluded that a higher TIL density was associated with poorer prognosis. For example, McArdle et al. performed a study in a cohort of 80 patients with PCa, in which they analyzed on whole tumor sections the infiltration of CD4^+^ and CD8^+^ lymphocytes within the tumor (including the cancer cell nests and surrounding stroma) but excluding any infiltration in the surrounding normal-like epithelium. The analysis revealed that a higher infiltration of CD4^+^ T lymphocytes in the tumor was associated with a shorter PCa specific-free survival in both univariate (HR= 2.03, 95% CI=1.15–3.59, p=0.015) and multivariate (HR=2.29, 95% CI=1.25-4.22, p=0.008) analyses. Infiltration by CD8^+^ T lymphocytes was however not predictive of PCa specific-free survival (21). A Norwegian study carried out on 535 primary PCa displayed on TMA, showed that in multivariate Cox regression analysis, a higher density of CD8^+^ lymphocytes in both tumor epithelial and tumor stromal areas taken as one single value, was an independent negative prognostic factor for BCR-free survival (HR= 1.565, CI 95%= 1.132–2.165, p= 0.007). Also, when the infiltration of CD8^+^ cells was assessed only in the tumor area, the association remained significant (HR=1.445, CI 95%1.028–2.032, p=0.032) (23). Flammiger et al. reported the analysis of a large TMA encompassing 3261 PCa samples. They found that both a low and high infiltration by CD3^+^ lymphocytes were associated with a shorter BCR‐free survival when compared with an intermediate level of infiltration in Kaplan-Meier analyses (p=0.019), a phenomenon known as the Goldilocks effect (24). This Goldilocks effect along with the study design including the characteristics of the tumor series, the choice of TMA *vs* whole sections to analyze the cell density in the different tumor compartments, the choice of the antibodies as well as the known heterogeneity of PCa could all contribute to generate variable results from one study to the other. Moreover, looking at the whole T lymphocyte population with markers such as CD3 or even CD4 and CD8 knowing that these cell populations are also heterogeneous as they contain subpopulations with anti-tumor, but also tumor-promoting phenotypes might not provide accurate prognosis. Looking at TIL corresponding to T cell subpopulation with more define characteristics such as T_reg_ and T_mem_ is expected to provide more informative data.

We showed in this study that the ratio of immune cells within the tumor area have important independent prognostic values. We showed that low ratio of FoxP3^+^/CD3^+^ or of FoxP3^+^/CD45RO^+^ cell density was associated with a lower risk of definitive ADT. Consistent with the previous finding, to the opposite a high ratio of FoxP3^+^/CD3^+^ cell density was associated with a higher risk of definitive ADT or lethal PCa and a high ratio of FoxP3^+^/CD45RO^+^ was associated with a higher risk of BCR. Our results are in concordance with results of other studies looking at the prognostic value of T_reg_. Davidsson et al. conducted a study using a TMA comprising tumor samples from 1367 men. The results inferred that neither infiltration of total CD4^+^ cells nor of CD8^+^ cells was associated with lethal PCa. However, a higher risk of lethal PCa was found when comparing the highest with the lowest quartile of FoxP3^+^ cells (odds ratio=1.98; 95% CI= 1.15–3.40) (25). Likewise, an Italian study aiming to characterize TIL in tumor and peripheral stroma areas of tumors of 22 men treated by radical prostatectomy and salvage radiotherapy concluded that a low infiltration by CD45^+^ and FoxP3^+^ cells in the peripheral stroma was correlated to a prolonged BCR-free survival and a better overall survival. However, there was no correlation between the infiltration by CD3^+^ and CD4^+^ TIL and clinical outcomes. Higher risks of dying from prostate PCa was found when comparing the highest with the lowest quartiles of FoxP3^+^ cells (odds ratio=1.98; 95% CI= 1.15–3.40) (26). Kaur et al. also found after analysis of the tumors of 144 African-American men an association between increased FoxP3^+^ cell density and a higher risk of metastasis in multivariate analysis (HR=12.89 (1.59-104.40) p=0.02) (33). More recently, Andersen et al. analyzed the prognostic potential of different immune cells in two large cohorts of radical prostatectomy specimens available in TMA (34). They observed that T_reg_ and M2 macrophages in stroma and epithelium, respectively, were adverse predictors of BCR in multivariate Cox regression analyses. A similar association of T_reg_ with BCR was found at the mRNA level in a third cohort therefore thus further supporting the association of T_reg_ with poorer prognosis (34). Using the same cohort as in this study, we recently reported the analysis of the prognostic value of the infiltration of tumors by macrophages and dendritic cells (DC) (46). We also found M2 macrophages were associated with a poorer prognosis. Indeed, a higher infiltration of CD163^+^ M2 macrophages in the normal adjacent epithelium as well as a higher infiltration of CD209^+^ immature DC at the tumor margin were associated with lethal PCa and BCR, respectively. Deeper analyses showed that the ratio of CD209^+^ immature DC over CD83^+^ mature DC was even more predictive of late adverse events, showing that the proportion of these immune cells must be taken into account to fully evaluate their prognostic value.

Our study also shows that CD45RO^+^ cell density and the ratio of CD45RO^+^/CD3^+^ cells in the tumor area was associated with good prognosis since a high density of CD45RO^+^ cells was associated with a lower risk of BCR and a high ratio of CD45RO^+^/CD3^+^ was associated with a lower risk of lethal PCa. To the contrary, a low ratio of CD45RO^+^/CD3^+^ density was associated with a higher risk of BCR. To our knowledge, our study appears to be the first to determine the prognostic value of CD45RO^+^ cells by immunohistochemistry in the microenvironment of PCa. There was one previous report of CD45RO^+^ cell infiltration in normal prostate but not in tumors (47). The infiltration of tumors by CD45RO^+^ cells was shown to be associated with clinical outcomes in various types of tumors (35, 48, 49). The independent prognostic value of CD45RO^+^ cells highlighted by this study underscore the importance to include this biomarker in future studies looking at TIL in PCa. Our data also show that the balance between CD45RO^+^ and FoxP3^+^ cells in the tumor area has an important prognostic value.

Supporting the results of the immunohistochemistry study, we found that high expression of FoxP3 gene as measured using TLDA technology was associated with a shorter lethal PCa-free survival in Kaplan-Meier analyses (**Figure 7**) but also in univariate Cox regression analysis (**Supplementary Table S4**). Amongst the genes we studied, CTLA-4 was the one that was the most highly correlated to FoxP3. The high expression of CTLA-4 by effector T_reg_ is well known and the use of anti-CTLA-4 is a relevant approach to kill T_reg_ or at least attenuate their suppressive activity (50–52). Beside CTLA-4, effector T_reg_ also express other ICP such as PD-1, TIM-3, LAG-3, GITR and OX40 that are also potential targets to modulate T_reg_ functions (50–53). In our analysis of the TLDA results we found that high expression of CTLA-4, PD-1, TIM-3 and LAG-3 genes together was associated with a shorter lethal PCa-free survival consistent with the immunosuppressive action of these molecules on T cells. However, when taken separately CTLA-4, PD-1 and LAG-3 were not significantly associated with the clinical outcomes but a high expression of TIM-3 was significantly associated with a shorter definitive ADT-free survival (**Figure 7**). Inhibition of TIM-3 might be an approach to consider to counteract the immunosuppressive activity of FoxP3^+^ cells in PCa as suggested by some authors (54–56).

This study has however some limitations. The first one concerns the technical approach. We used in this study a standard IHC technique in which each slide is stained with a single antibody against a marker specific to a cell population instead of performing a multiplex analysis for the simultaneous detection of different markers and corresponding cell populations on a single slide. We selected the standard IHC approach for practical reasons and to ease an eventual clinical application as this approach is still the most frequently used in clinical pathology laboratories. However, when the objective is to determine a ratio between cell populations, this approach is less adapted and tedious compared to the use of a multiplex assay. Moreover, the multiplex assay also have the advantage to detect various cell phenotypes i.e. cell expressing combinations of markers. For example, it was reported that some T_reg_ cells may also express CD45RO (57–59). The use of a multiplex assay would allow to assess the prognostic value of the density of such double positive FoxP3^+^CD45RO^+^ cell population in addition of that of the single positive FoxP3^+^ or CD45RO^+^ cell populations. In this study, we could not detect this double positive cell population and so we don’t know the frequency of these cells in the series of tumors we analyzed. But since the FoxP3^+^ cell population is about 8 times less numerous than the CD45RO^+^ cell population, the occurrence of some cells expressing both markers is not expected to significantly modify the conclusion of the study. A second limitation of the study is the size of the cohorts. In several occasions, association with the outcomes did not reach statistical significance because of the limited size of the cohorts. The results of this study should be validated in larger cohorts.

In conclusion, we showed in this study that the infiltration of the tumor area by FoxP3^+^ T_reg_ and CD45RO^+^ T_mem_ cells are highly predictive of late clinical events when they are related to the CD3^+^ cell population or related to one another using ratio of cell density. Using ratio of FoxP3^+^ and CD45RO^+^ over CD3^+^ cell density or to one another is a way to normalize the frequency of these cells in the tissue which hopefully might offer more reproducible results. Our gene expression analysis by TLDA supported the association between a high expression of *FoxP3* gene and a higher risk of lethal PCa. It also identified a correlation between a high TIM-3 gene expression with a higher risk for definitive ADT. These results let us to suggest that inhibition of TIM-3 might be a relevant approach to counter the immunosuppressive functions of T_reg_ in order to improve the anti-tumor immune response against PCa.

## 4 Material and methods

### 4.1 Patient data and tissue samples

Two cohorts of patients treated by radical prostatectomy at CHU de Québec-Université Laval were used for this study (**Figure 1**). The first cohort (IHC cohort) was composed of 98 men treated between March 1996 and November 1998. This cohort is composed of men that had a least one factor that increased their chance to experience progression, i.e. an extraprostatic extension, a positive margin, a lymph node invasion or a high-grade tumor. For each participant, whole sections of a tissue block representative of the tumor but also containing normal-like adjacent epithelium were used for IHC analyses. The second cohort (TLDA cohort) was composed of 50 men who underwent radical prostatectomy between September 2004 and August 2009. Tumor tissues frozen in optimal cutting temperature (OCT) compound were available for this cohort and were used for the gene expression analysis using TLDA. Clinico-pathological data used include patient demographics, tumor, BCR, ADT, development of metastases and survival data. Time to lethal PCa was defined as either death from PCa and/or occurrence of metastasis and/or development of CRPC status. Further details on the definition of the clinical outcomes used in this study are presented in the **Supplementary Material and Methods**.

### 4.2 Immunohistochemistry

The most representative formalin-fixed and paraffin-embedded (FFPE) tumor block was cut to prepare consecutive 5 µm-thick sections which were dried overnight at 37°C. Sections were deparaffinized and heat-induced antigen retrieval was performed using a PT Link (Pre-Treatment Module for Tissue Specimens) with either citrate buffer pH 6.1 (Dako Code K8005: EnVision™ FLEX, Low pH) for CD3 and CD45RO or Tris/EDTA, pH 9 (Dako Code K8004: EnVision™ FLEX, High pH) for FoxP3. The immunodetection was performed using the IDetect Super Stain HRP-Polymer kit (ID labs, London, Ontario, Canada) after blockade of endogenous peroxidase activity by incubation in 3% peroxide solution for 10 min. Briefly, slides were incubated with Super block solution for 10 min to prevent non-specific background. Sections were then incubated overnight at room temperature with primary antibodies against CD3 (clone SP7, dilution 1/500, Abcam, Toronto, ON), CD45RO (clone UCHL-1, dil 1/6000, Abcam), and FoxP3 (clone 236A/E7, dil 1/600, Abcam). After washes, slides were incubated for 30 min with HRP-Polymer Conjugate. After 5 min of staining with DAB (3,3′-Diaminobenzidine), the slides were rinsed, counterstained with hematoxylin, dehydrated and mounted using MM 24 low viscosity mounting medium (Leica Microsystems, Durham, USA). Slides were digitalized using a Nanozoomer (Hamamatsu Photonics, Bidgewater NJ, USA) and visualized using the NDP.view2 software (Hamamatsu Photonics).

For each section, ten fields of view at 20x magnification (surface area of 0.460 mm^2^) were randomly selected in the tumor, tumor margin and normal-like areas. The number of positive cells in each field of view was determined either manually by two trained observers (OEM and HL) or by a trained observer (OEM) and semi-automatically using the Calopix software (TRIBVN Healthcare, Châtillon, France). For each marker, 10% of the slides were randomly selected then reviewed and confirmed by a trained pathologist (BT).

### 4.3 Gene expression analysis

For these analyses, frozen tumor specimens were selected to ensure representation of normal-like tissues and tumor margins, so we selected specimens in which tumor area represented between 30 and 70% of the whole tissue. We also used, as control tissue for gene expression normalization, six normal prostate tissues from cadaver organ donors that had no PCa after pathology review. For each of the 50 eligible tumors, ten slides of 10 μm in thickness were used for RNA extraction. An H&E-stained section was prepared before and after the ten sections for RNA extraction to ensure that the tumor was present throughout the tissue depth. RNA extraction was performed on the ten frozen tumor sections using the Quick mirVana™ miRNA Isolation Kit from Ambion (ThermoFisher Scientific) in accordance with the manufacturer’s instructions. Following extraction, DNA contamination was removed from the RNA samples using the Ambion DNAfree™ kit (ThermoFisher Scientific) in accordance with the manufacturer’s instructions. The RNA was then reverse transcribed into first strand cDNA using the SuperScript^®^ VILO™ Invitrogen system (Life technologies, Waltham, MA, USA). TaqMan^®^ Array Micro Fluidic 384-Wells TLDA cards (Life technologies) were custom designed with pre-loaded gene-specific primer and probe sets for the analysis of 31 selected immune gene targets and two house-keeping genes for mRNA normalization (**Supplementary Table S3**). Each cDNA sample, 300 ng at a concentration of 3 ng/μl were added to an equal volume of 2X TaqMan Universal PCR Master Mix (Thermo Scientific) and 100 µL of the sample-specific PCR mix was added to the fill reservoir on the TLDA card. The card was centrifuged twice for one minute at 1200 rpm and sealed using the TaqMan Array Micro Fluidic Card Sealer (Thermo Scientific). The amplification was performed in a StepOnePlus™, 7900HT Fast Real-Time PCR System (Applied Biosystems) using the following cycling conditions: 2 min at 50°C, 10 min at 94.5°C, 30 s at 97°C, 1 min at 59.7°C for 40 cycles. The mRNA expression levels were normalized to GUSB and PPIA (reference genes), and the expression values of immune gene expression were calculated using ΔΔCT method, as recommended by the manufacturer. Each tumor gene expression value was then reported as a fold change of the same gene mean value in normal prostates. This resulting value was used for statistical analysis.

### 4.4 Statistics

The characteristics of the patients in each cohort are summarized by means, standard deviation ( ± SD), frequency, and percentage. Time-to-event period for each outcome was calculated from the date of surgery to corresponding event date or to last follow-up date, for right censored cases. Univariate and multivariate Cox proportional hazards regression models were used to estimate the hazard ratio (HR) and HR adjusted for age, PSA, Gleason grade, T (tumor stage), N (nodal stage), and margin status. The assumption of proportional hazards was evaluated using the supreme test for all Cox regression models. Kaplan-Meier curves of markers categorized in either quartiles (or tertiles) and dichotomized by higher or lower quartiles (or tertiles) were used to estimate the association with clinical outcomes and the log-rank test was used to assess the differences between the curves. Pearson correlations (*r_p_*) were used to estimate the correlation between gene expression targets. Statistical analyses were performed using SAS Statistical Software v.9.4 (SAS Institute, Cary, NC, USA), with a two-sided significance level set at p ≤ 0.05.

## Data availability statement

The raw data supporting the conclusions of this article will be made available by the authors, without undue reservation.

## Ethics statement

The studies involving humans were approved by Research ethics committee of the CHU de Québec-Université Laval. The studies were conducted in accordance with the local legislation and institutional requirements. The participants provided their written informed consent to participate in this study.

## Author contributions

OM: Visualization, Validation, Methodology, Investigation, Formal analysis, Data curation, Conceptualization, Writing – review & editing, Writing – original draft. HL: Writing – review & editing, Supervision, Resources, Project administration, Methodology, Investigation, Funding acquisition, Formal analysis, Data curation, Conceptualization. DS: Writing – review & editing, Methodology, Formal analysis. HH: Writing – review & editing, Resources, Investigation. BV: Writing – review & editing, Investigation, Formal analysis. BT: Writing – review & editing, Investigation, Funding acquisition. VF: Writing – review & editing, Funding acquisition. LL: Writing – review & editing, Methodology, Funding acquisition, Conceptualization. AB: Writing – review & editing, Writing – original draft, Supervision, Resources, Funding acquisition. YF: Writing – review & editing, Writing – original draft, Supervision, Resources, Project administration, Methodology, Funding acquisition, Formal analysis, Conceptualization.

## Supplementary material

Supplementary Figure 1 Example of a prostate cancer sample with delimitation of normal-like epithelium, tumor margin and tumor areas. For scoring of each slide after staining with the different antibodies, ten fields of view at 20x magnification (surface area of 0.460 mm^2^) were randomly selected in the tumor (represented as red rectangles in the red-encircled zone), tumor margin (represented as black rectangles at the periphery of the tumor) and normal-like areas (represented as green rectangles in the green-encircled zones). The number of positive cells in each field of view was determined either manually by two trained observers or by a trained observer and semi-automatically using the Calopix software (TRIBVN Healthcare, Châtillon, France). Magnification 4X.

Supplementary Figure 2 Immunohistochemical analysis of the infiltration by T lymphocytes. **(A-J)** Examples of staining for CD3^+^, CD45RO^+^ and FOXP3^+^ cells in normal-like adjacent epithelium, the tumor margin and in the tumor areas. Magnification 20X. Insert magnification equivalent to 40X. Scale bar = 200 µm. **(K)** The mean number of CD3^+^, CD45RO^+^, or FoxP3^+^ cells ± SD per mm^2^ infiltrating the adjacent normal-like adjacent epithelium, tumor margin and tumor areas is provided in the table. SD: Standard deviation.

Supplementary Table 1 Multivariate Cox regression analysis calculated HR according to the three clinical outcomes.

Supplementary Table 2 Multivariate Cox regression analysis calculated HR to predict the risk for each clinical outcomes according to low (Q1 *vs* Q2-Q4) or high (Q4 *vs* Q1-Q3) ratio of the number of cells infiltrating the various tissue compartments. HR was adjusted for age, PSA, Gleason grade, T stage, N stage, and margin status.

Supplementary Table 3 List of immune genes analyzed in TLDA experiment and their associated activity or pathway.

Supplementary Table 4 Un**i**variate Cox regression analysis calculated HR to predict the risk for each clinical outcomes according to a high (T3 *vs* T1-T2) level of expression of FoxP3, CTLA-4 (CTLA4), TIM-3 (HAVCR2), LAG-3 (LAG3) and PD-1 (PDCD1) genes.
