# Supplementary material for: Regulatory and memory T lymphocytes infiltrating prostate tumors predict long term clinical outcomes
Source: Front Immunol. 2024 Jun 3;15:1372837. doi: 10.3389/fimmu.2024.1372837 (PMC11180786; doi:10.3389/fimmu.2024.1372837)
Supplement: Supplementary Figure 1 — Example of a prostate cancer sample with delimitation of normal-like epithelium, tumor margin and tumor areas. For scoring of each slide after staining with the different antibodies, ten fields of view at 20x magnification (surface area of 0.460 mm2) were randomly selected in the tumor (represented as red rectangles in the red-encircled zone), tumor margin (represented as black rectangles at the periphery of the tumor) and normal-like areas (represented as green rectangles in the green-encircled zones). The number of positive cells in each field of view was determined either manually by two trained observers or by a trained observer and semi-automatically using the Calopix software (TRIBVN Healthcare, Châtillon, France). Magnification 4X. [file DataSheet_1.pdf]

Supplementary Material

Regulatory and memory T lymphocytes infiltrating prostate tumors predict long term clinical outcomes

Oscar Eduardo Molina[1,2], Hélène LaRue[1,2], David Simonyan[3], Hélène Hovington[1,2], Benjamin Vittrant[1,2], Bernard Têtu[1,2,4], Vincent Fradet[1,2,5], Louis Lacombe[1,2,5], Alain Bergeron[1,2,5], Yves Fradet*[1,2,5]

[1]Axe oncologie, Centre de recherche du CHU de Québec-Université Laval, Québec, Canada, [2]Centre de recherche sur le cancer de l'Université Laval, Québec, Canada, [3]Plateforme de recherche clinique et évaluative, Centre de recherche du CHU de Québec-Université Laval, Québec, Canada, [4]Département de pathologie, CHU de Québec-Université Laval, Québec, Canada, [5]Département de chirurgie de l'Université Laval, Québec, Canada.

*** Correspondence:**
Yves Fradet
yves.fradet@crchudequebec.ulaval.ca

1 Supplementary Material and Methods

1.1 Clinical outcome definition

The main clinical endpoints used in this project are the biochemical recurrence, definitive androgen deprivation therapy and the lethal prostate cancer. Below is the definition of each of these clinical outcomes.

1.1.1 Biochemical recurrence (BCR)

In this study, we defined the biochemical recurrence of prostate cancer as any of the following scenarios.

-Two consecutives PSA measured levels of 0.3 ng/mL or greater after the radical prostatectomy.

-One PSA measured level of 0.3 ng/mL or greater followed by a hormonal or a radiotherapy treatment after the radical prostatectomy.

-Any PSA measured level lower than 0.3 ng/mL followed by a hormonal or a radiotherapy treatment after the radical prostatectomy.

-Also, the failure to the radical prostatectomy, defined as any case where the PSA level doesn't reach a value of zero (in this case the limit of detection is 0.01 ng/mL) 3 months after the surgery.

1.1.2 Definitive androgen deprivation therapy (ADT)

In this study, we defined definitive androgen deprivation therapy as any type of hormonal therapy other than the neoadjuvant and/or adjuvant to a primary therapy that is carried out for life or until sings of disease progression**.**

1.1.3 Lethal prostate cancer (PCa)

Lethal PCa is defined as castrate resistant prostate cancer (CRPC) and/or metastasis and/or PCa specific death. Once the patient is diagnosed with CRPC or metastasis, there is no chance of curative treatment possible, and those patients are in a path where they will eventually die from cancer (unless another cause of death occurs before). CRPC, in this study, is defined as a disease progression despite definitive hormonal therapy and may be confirmed by either a continuous rise in serum PSA levels, the progression of pre-existing disease, and/or the appearance of new metastases. Metastasis in PCa is defined as the development of secondary malignant growths at a distance from prostate. PCa specific death is define as the death of patient associated to the disease progression, in this category we exclude all other causes deaths.

1.2 SUPPLEMENTARY RESULTS

Qualitative description of the staining for the three types of cells.

1.2.1 CD3$^+$ cells – T lymphocytes

The peritumoral areas display a heterogeneous infiltration pattern with rounded CD3$^+$ infiltrating lymphocytes, usually found both beside the basal cells and intercalated between the normal epithelial cells. In the tumor areas, the CD3$^+$ infiltrating lymphocytes are rounded and heavily stained, most of the infiltration is found in the tumor stroma. Finally, the infiltration in the tumor margin is high and exhibiting a slightly different pattern than the other areas. At the tumor margin the cells are most likely found towards the peritumoral areas.

1.2.2 CD45RO$^+$ cells - Memory T lymphocytes

The infiltration by CD45RO$^+$ cells in the tumor and peritumoral areas is characterized by a heterogeneous distribution with most of the CD45RO$^+$ lymphocytes found principally beside the basal cells of the normal glands and clusters of CD45RO$^+$ cells scatter in the stroma. The tumor margin is the most infiltrated area, the distribution is somehow homogeneous, and the infiltrating cells are found more often near the peritumoral limit of the invasive margin.

1.2.3 FoxP3$^+$ cells - Regulatory T lymphocytes

Infiltration by FoxP3$^+$ cells in the peritumoral area is low, heterogeneous, and centered around the areas close to the basal cell of the normal glands. The tumor areas are highly infiltrated by small cells with a rounded morphology found around the tumor glands near to the base of the tumor epithelium. The tumor margin infiltration is heterogeneous, the majority of FoxP3$^+$ cells are round, small, heavily stained and found in the tumor limit of the invasive margin.

2 Supplementary Figure and Tables

2.1 Supplementary Figures

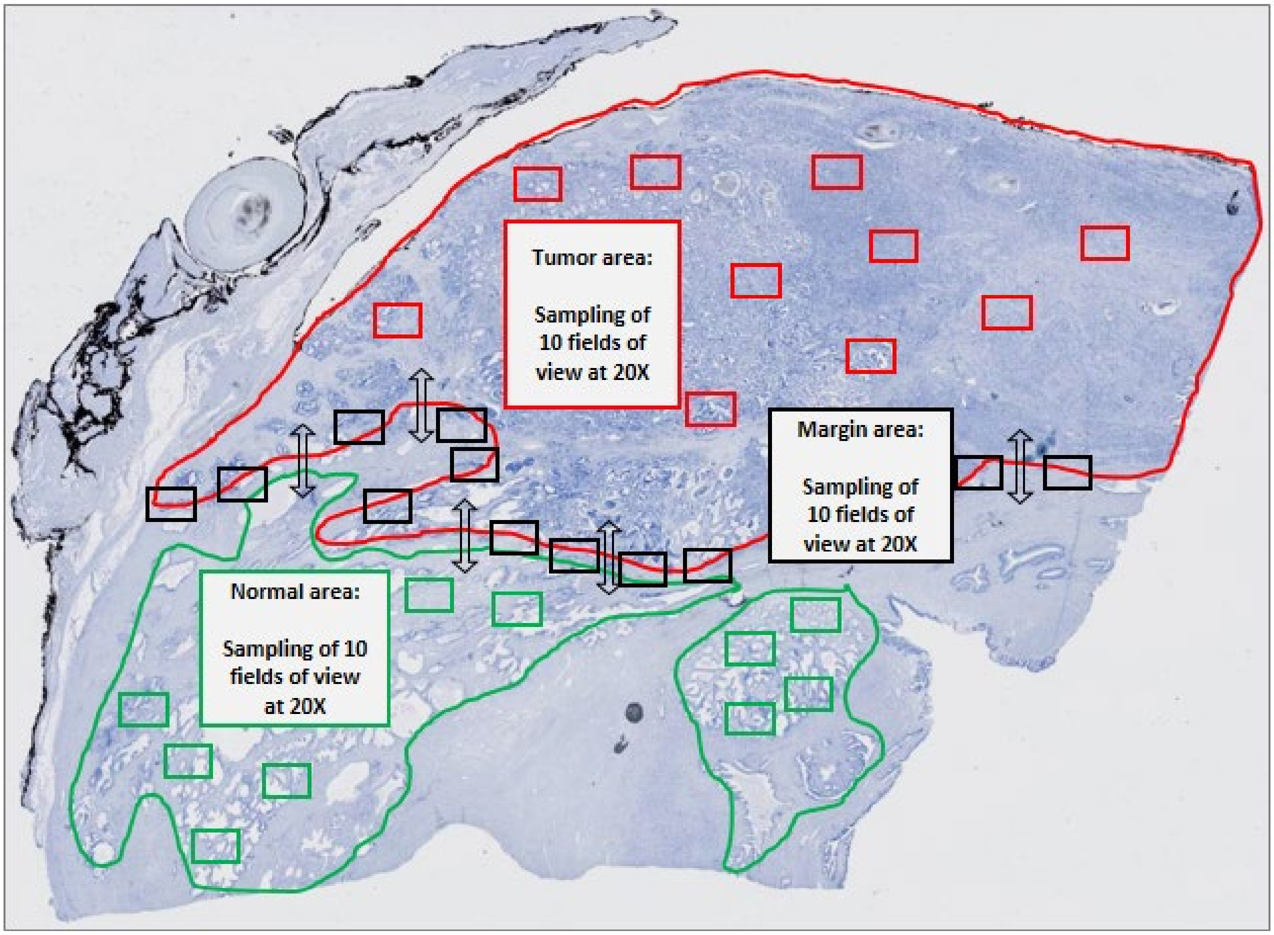

Supplementary Figure S1. Example of a prostate cancer sample with delimitation of normal-like epithelium, tumor margin and tumor areas. For scoring of each slide after staining with the different antibodies, ten fields of view at 20x magnification (surface area of 0.460 mm^2) were randomly selected in the tumor (represented as red rectangles in the red-encircled zone), tumor margin (represented as black rectangles at the periphery of the tumor) and normal-like areas (represented as green rectangles in the green-encircled zones). The number of positive cells in each field of view was determined either manually by two trained observers or by a trained observer and semi-automatically using the Calopix software (TRIBVN Healthcare, Châtillon, France). Magnification 4X.

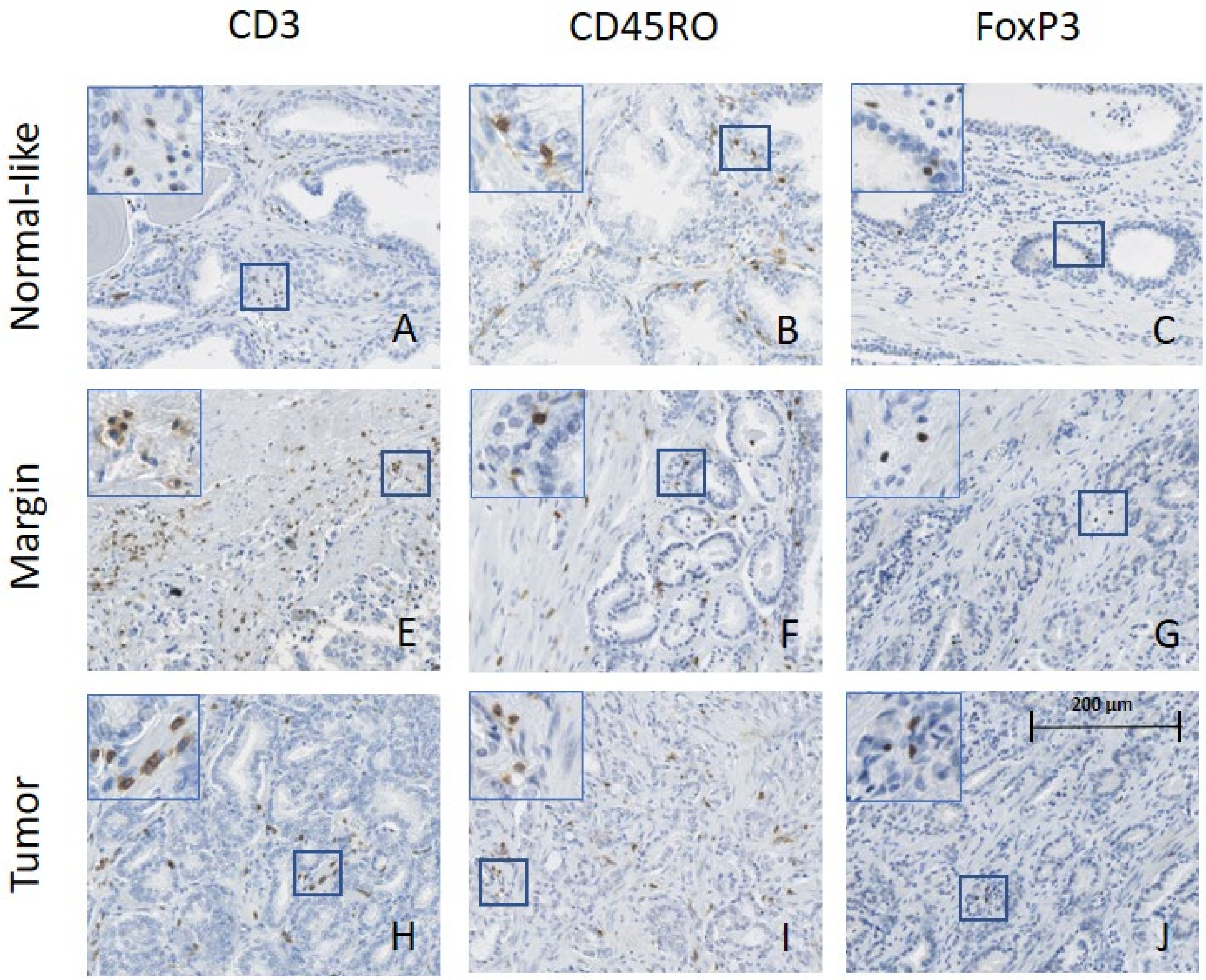

K

	Mean number of CD3⁺ cell ± SD/mm²	Mean number of CD45RO⁺ cell ± SD/mm²	Mean number of FoxP3⁺ cell ± SD/mm²
Normal-like	74.3 ± 49.7	60.8 ± 46.7	7.1 ± 7.6
Margin	112.8 ± 102.9	80.1 ± 52.7	10.1 ± 7.9
Tumor	72.8 ± 35.8	73.3 ± 48.9	9.3 ± 7.6

Supplementary Figure S2. Immunohistochemical analysis of the infiltration by T lymphocytes. A-J) Examples of staining for $CD3^+$, $CD45RO^+$ and $FOXP3^+$ cells in normal-like adjacent epithelium, the tumor margin and in the tumor areas. Magnification 20X. Insert magnification equivalent to 40X. Scale bar = 200 µm. K) The mean number of $CD3^+$, $CD45RO^+$, or $FoxP3^+$ cells ± SD per mm^2 infiltrating the adjacent normal-like adjacent epithelium, tumor margin and tumor areas is provided in the table. SD: Standard deviation.

2.2 Supplementary Tables

Supplementary Table S1. IHC cohort: Multivariate Cox regression analysis calculated hazard ratios (HR) according to the three clinical outcomes.

			BCR		Definitive ADT		Lethal PCa	
**Parameter**	**n**	**%**	**HR (95% CI)**	***p* value**	**HR (95% CI)**	***p* value**	**HR (95% CI)**	***p* value**
**Age (mean ± SD)**								
63.4 ± 5.2 yrs	98	100.0	0.98 (0.92-1.04)	0.411	0.96 (0.89-1.04)	0.298	1.01 (0.93-1.10)	0.877
**PSA**								
≤10 ng/mL (ref)	54	55.1	1		1		1	
10 - 20 ng/mL	29	29.6	1.37 (0.67-2.79)	0.394	2.13 (0.74-6.15)	0.162	0.65 (0.17-2.45)	0.524
≥20 ng/mL	15	15.3	1.49 (0.65-3.40)	0.350	1.40 (0.44-4.47)	0.567	1.02 (0.29-3.64)	0.971
**Gleason Group Grade**								
1 (Gleason 6)	22	22,5	0.64 (0.25-1.65)	0.358	-	-	-	-
2 (Gleason 3+4) (ref)	35	35.7	1		1		1	
3 (Gleason 4+3)	17	17.3	1.40 (0.61-3.19)	0.428	0.79 (0.23-2.66)	0.703	2.83 (0.65-12.35)	0.166
4/5 (Gleason ≥8)	24	24.5	1.27 (0.58-2.76)	0.552	1.57 (0.55-4.46)	0.398	2.24 (0.67-7.53)	0.193
**T Stage**								
pT2	33	33.7	0.53 (0.23-1.21)	0.132	2.41 (0.57-10.23)	0.233	0.80 (0.07-9.14)	0.858
pT3a (ref)	33	33.7	1		1		1	
pT3b/pT4	32	32.6	1.73 (0.83-3.61)	0.149	**4.18 (1.16-15.10)**	**0.029**	**6.40 (1.35-30.41)**	**0.020**
**N Stage**								
negative (ref)	70	71.4	1		(-)		1	
positive	28	28.6	0.85 (0.43-1.70)	0.647	**4.28 (1.61-11.37)**	**0.004**	**3.30 (1.07-10.16)**	**0.038**
**Margin**								
negative (ref)	22	22.4	1		(-)		1	
positive	76	77.6	1.52 (0.72-3.21)	0.271	0.73 (0.26-2.04)	0.546	5.16 (0.96-27.58)	0.055

Supplementary Table S2. Multivariate Cox regression analysis calculated hazard ratios (HR) to predict the risk for each clinical outcomes according to low (Q1 vs Q2-Q4) or high (Q4 vs Q1-Q3) ratio of the number of cells infiltrating the various tissue compartments. HR was adjusted for age, PSA, Gleason grade, T stage, N stage, and margin status.

**Outcome/ Marker**	**Low Ratio**		**High Ratio**	
	**HR (95% CI)**	***p* value**	**HR (95% CI)**	***p* value**
**BCR**				
$FoxP3/CD3_N$	0,93 (0,43-2,02)	0,850	1,42 (0,65-3,13)	0,377
$FoxP3/CD3_M$	0,83 (0,37-1,89)	0,655	0,99 (0,44-2,23)	0,986
$FoxP3/CD3_T$	1,06 (0,49-2,27)	0,885	0,92 (0,45-1,89)	0,829
$CD45RO/CD3_N$	1,32 (0,63-2,77)	0,468	0,58 (0,25-1,38)	0,221
$CD45RO/CD3_M$	1,16 (0,57-2,35)	0,691	0,91 (0,43-1,90)	0,796
**$CD45RO/CD3_T$**	**2,18 (1,15-4,13)**	**0,017**	0,74 (0,34-1,61)	0,447
$FoxP3/CD45RO_N$	1,05 (0,45-2,43)	0,919	0,93 (0,44-2,00)	0,862
$FoxP3/CD45RO_M$	1,15 (0,50-2,65)	0,743	1,61 (0,78-3,32)	0,201
$FoxP3/CD45RO_T$	0,51 (0,22-1,16)	0,109	**2,54 (1,25-5,18)**	**0,010**
**Defenitive ADT**				
$FoxP3/CD3_N$	0,88 (0,24-3,15)	0,838	0,68 (0,22-2,11)	0,505
$FoxP3/CD3_M$	0,33 (0,07-1,58)	0,163	1,75 (0,57-5,35)	0,328
**$FoxP3/CD3_T$**	**0,10 (0,02-0,50)**	**0,006**	**7,69 (2,27-25,64)**	**0,001**
$CD45RO/CD3_N$	0,65 (0,19-2,26)	0,501	1,47 (0,42-5,21)	0,549
$CD45RO/CD3_M$	0,60 (0,19-1,91)	0,384	0,97 (0,27-3,46)	0,958
$CD45RO/CD3_T$	0,71 (0,25-2,02)	0,521	0,64 (0,15-2,73)	0,552
$FoxP3/CD45RO_N$	0,85 (0,19-3,85)	0,836	0,17 (0,03-1,02)	0,052
$FoxP3/CD45RO_M$	0,15 (0,02-1,29)	0,084	1,28 (0,40-4,08)	0,676
**$FoxP3/CD45RO_T$**	**0,10 (0,02-0,53)**	**0,007**	1,98 (0,69-5,62)	0,202
**Lethal PCa**				
$FoxP3/CD3_N$	0,28 (0,06-1,35)	0,112	3,98 (0,95-16,67)	0,059
$FoxP3/CD3_M$	0,43 (0,07-2,59)	0,358	0,89 (0,26-3,04)	0,858
$FoxP3/CD3_T$	0,25 (0,04-1,37)	0,110	**4,37 (1,07-17,86)**	**0,040**
$CD45RO/CD3_N$	1,07 (0,27-4,31)	0,921	2,15 (0,44-10,64)	0,347
$CD45RO/CD3_M$	1,06 (0,29-3,84)	0,927	0,64 (0,14-2,95)	0,564
$CD45RO/CD3_T$	1,29 (0,40-4,14)	0,673	**0,06 (<0.01-0,87)**	**0,040**
$FoxP3/CD45RO_N$	0,95 (0,11-8,08)	0,962	0,38 (0,06-2,59)	0,325
$FoxP3/CD45RO_M$	0,17 (0,01-2,13)	0,169	0,74 (0,18-3,06)	0,673
$FoxP3/CD45RO_T$	0,17 (0,03-1,06)	0,058	1,43 (0,42-4,85)	0,568

N= adjacent normal-like epithelium, M= tumor margin and T= tumor area.

Supplementary Table S3. List of immune genes analyzed in TLDA experiment and their associated usual activity or pathway.

Gene-Assay ID[1]	Gene Name	Cell type or molecule type[2]
CD8A-Hs00233520_m1	CD8a molecule	CTL
GNLY-Hs00246266_m1	Granulysin	CTL
GZMB-Hs01554355_m1	Granzyme B	CTL
PRF1-Hs00169473_m1	Perforin 1 (pore forming protein)	CTL
CCL24-Hs00171082_m1	Chemokine (C-C motif) ligand 24 (eotaxin-2)	Eosinophils/T cells
CD247-Hs00609515_m1	CD247 molecule (CD3 zeta chain)	T cells
CD2-Hs00233515_m1	CD2 molecule	T cells
CD3G-Hs00962186_m1	CD3g molecule, gamma (CD3-TCR complex)	T cells
CD4-Hs01058407_m1	CD4 molecule	T cells
IL2RB-Hs01081697_m1	Interleukin 2 receptor, beta	T cells
IFNG-Hs00989291_m1	Interferon, gamma	Th1
IRF1-Hs00971960_m1	Interferon regulatory factor 1	Th1
STAT4-Hs01028017_m1	Signal transducer and activator of transcription 4	Th1
TAP1-Hs00388675_m1	Transporter 1, ATP-binding cassette, sub-family B (MDR/TAP)	Th1
TBX21-Hs00203436_m1	T-box 21	Th1
TNF-Hs01113624_g1	Tumor necrosis factor	Th1
IL17A-Hs00174383_m1	Interleukin 17A	Th17
RORC-Hs01076122_m1	RAR-related orphan receptor C	Th17
CD40LG-Hs00163934_m1	CD40 ligand	Th2
GATA3-Hs00231122_m1	GATA binding protein 3	Th2
IL13-Hs00174379_m1	Interleukin 13	Th2
IL4-Hs00174122_m1	Interleukin 4	Th2
SMAD2-Hs00183425_m1	SMAD family member 2	Th2
STAT6-Hs00598625_m1	Signal transducer and activator of transcription 6	Th2
FOXP3-Hs01085834_m1	Forkhead box P3	Treg
IL10-Hs00961622_m1	Interleukin 10	Treg
TGFB1-Hs00998133_m1	Transforming growth factor, beta 1	Treg
CTLA4-Hs03044418_m1	Cytotoxic T-lymphocyte-associated protein 4	Treg/ICP
HAVCR2-Hs00958618_m1	Hepatitis A virus cellular receptor 2 (TIM3)	ICP
LAG3-Hs00158563_m1	Lymphocyte-activation gene 3	ICP
PDCD1-Hs01550088_m1	Programmed cell death 1	ICP

CTL: Cytotoxic T lymphocytes; Th1: T helper 1; Th2: T helper 2; Th17: T helper 17; Treg: regulatory T cells; ICP: Immune checkpoint

[1] Gene symbol and Assay ID from ThermoFisher Scientific

[2] See Bindea *et al.* Immunity *39*, 782-795, 2013 and GeneCards.org

Supplementary Table S4. Univariate Cox regression analysis calculated hazard ratios (HR) to predict the risk for each clinical outcomes according to a high (T3 vs T1-T2) level of expression of FoxP3, CTLA-4 (CTLA4), TIM-3 (HAVCR2), LAG-3 (LAG3) and PD-1 (PDCD1) genes.

**Outcome / Marker**	**HR (95% CI)**	***p* value**
**BCR**		
FoxP3	1,75 (0,83-3,68)	0,144
CTLA-4	1,52 (0,72-3,21)	0,268
TIM-3	1,34 (0,63-2,84)	0,447
LAG-3	1,22 (0,57-2,58)	0,611
PD-1	0,98 (0,44-2,19)	0,964
**Definitive ADT**		
FoxP3	2,05 (0,83-5,05)	0,120
CTLA-4	1,36 (0,55-3,39)	0,504
**TIM-3**	**3,11 (1,25-7,69)**	**0,014**
LAG-3	1,34 (0,52-3,40)	0,544
PD-1	1,40 (0,54-3,64)	0,485
**Lethal PCa**		
**FoxP3**	**5,26 (1,35-20,41)**	**0,017**
CTLA-4	1,85 (0,53-6,45)	0,337
TIM-3	2,49 (0,72-8,62)	*0,150*
LAG-3	1,44 (0,40-5,13)	0,575
PD-1	1,68 (0,46-6,06)	0,430
